# Progressive Photoreceptor Dysfunction and Age-Related Macular Degeneration-Like Features in *rp1l1* Mutant Zebrafish

**DOI:** 10.3390/cells9102214

**Published:** 2020-09-30

**Authors:** Nicole C. L. Noel, Nathan J. Nadolski, Jennifer C. Hocking, Ian M. MacDonald, W. Ted Allison

**Affiliations:** 1 Department of Medical Genetics, University of Alberta, Edmonton, AB T6G 2H7, Canada; nadolski@ualberta.ca (N.J.N.); jhocking@ualberta.ca (J.C.H.); macdonal@ualberta.ca (I.M.M.); ted.allison@ualberta.ca (W.T.A.); 2 Division of Anatomy, Department of Surgery, University of Alberta, Edmonton, AB T6G 2H7, Canada; 3 Department of Cell Biology, University of Alberta, Edmonton, AB T6G 2H7, Canada; 4 Women and Children’s Health Research Institute, University of Alberta, Edmonton, AB T6G 1C9, Canada; 5 Department of Ophthalmology and Visual Sciences, University of Alberta, Edmonton, AB T6G 2R7, Canada; 6 Department of Biological Sciences, University of Alberta, Edmonton, AB T6G 2E9, Canada; 7 Centre for Prions and Protein Folding Diseases, University of Alberta, Edmonton, AB T6G 2M8, Canada

**Keywords:** rp1l1, axoneme, maculopathy, photoreceptor outer segment, occult macular dystrophy, retinitis pigmentosa, retinal dystrophy, age-related macular degeneration, subretinal drusenoid deposits, drusen

## Abstract

Photoreceptor disease results in irreparable vision loss and blindness, which has a dramatic impact on quality of life. Pathogenic mutations in *RP1L1* lead to photoreceptor degenerations such as occult macular dystrophy and retinitis pigmentosa. RP1L1 is a component of the photoreceptor axoneme, the backbone structure of the photoreceptor’s light-sensing outer segment. We generated an *rp1l1* zebrafish mutant using CRISPR/Cas9 genome editing. Mutant animals had progressive photoreceptor functional defects as determined by electrophysiological assessment. Optical coherence tomography showed gaps in the photoreceptor layer, disrupted photoreceptor mosaics, and thinner retinas. Mutant retinas had disorganized photoreceptor outer segments and lipid-rich subretinal drusenoid deposits between the photoreceptors and retinal pigment epithelium. Our mutant is a novel model of *RP1L1*-associated photoreceptor disease and the first zebrafish model of photoreceptor degeneration with reported subretinal drusenoid deposits, a feature of age-related macular degeneration.

## 1. Introduction

Photoreceptors are sensory neurons that detect light via an elaborated cilium, called the outer segment (OS). The photoreceptor OS contains membranous discs densely packed with opsins, the photosensitive proteins. As the outer segment is a modified cilium, pathogenic variants of ciliary components are common causes of photoreceptor disease [1,2]. The morphology of the outer segment distinguishes the two types of photoreceptors, cones and rods. Cones allow for high-acuity daytime and color vision, while rods are responsible for low-light vision. The human retina is organized such that the peripheral retina is rod dense and the central retina, termed the macula, is cone dense. Diseases that lead to the degeneration or dysfunction of the cone photoreceptors in the macula are called maculopathies.

The most common maculopathy is age-related macular degeneration (AMD). AMD is the leading cause of vision loss for individuals over 50 in North America, affecting ~2% of people [3]. Patients with AMD experience visual acuity decline, color vision deficits, and central vision loss. There are two types of AMD: dry and wet AMD. Dry AMD is characterized by the accumulation of drusen and subretinal drusenoid deposits, which are lipid-rich deposits that occur in specific retinal locations. Drusen collect between the retinal pigment epithelium (RPE) and underlying choroid vasculature, while subretinal drusenoid deposits build up between the photoreceptor OSs and the RPE. In advanced cases, dry AMD can progress to wet AMD, characterized by retinal neovascularization. Neovascularization can lead to the leakage of blood into the retina and further tissue damage. Despite AMD being a common cause of vision loss, there are no effective treatments for dry AMD and only angiogenesis inhibitors for blocking aberrant vessel growth in wet AMD. The lack of therapeutics results in part from challenges surrounding the generation of animal models to investigate the disease mechanisms underlying AMD progression. AMD is a multifactorial condition, with many genetic and environmental contributors, making it difficult to establish a genetic model. Additionally, commonly used animal models for studying human disease, such as mice and rats, have minimal cones and no macula and rarely exhibit many of the hallmark features of AMD during retinal degeneration progression, like drusen or subretinal drusenoid deposits.

Occult macular dystrophy (OMD) is a rare inherited maculopathy. Similar to AMD, OMD is characterized by progressive central vision loss and color vision disturbances [4,5,6]. OMD was initially termed “occult” because OMD patients had normal appearing maculae, as observed by clinical fundus imaging, despite central vision loss [4,6]. Modern optical coherence tomography (OCT) imaging revealed that OMD patients do in fact exhibit loss of macular photoreceptors and retinal thinning [7]. Recently, it was reported that OMD patients can progress to a maculopathy with fundus findings [8].

OMD is caused by mutations in the gene *Retinitis Pigmentosa 1-Like 1* (*RP1L1*) [9]. RP1L1 is a component of the photoreceptor axoneme [10,11,12]. It is a microtubule-binding protein unique to photoreceptors [11]. The function of RP1L1 in the axoneme is unknown, although it is believed to bind the axoneme microtubules and associate with other (currently unidentified) proteins [8,10,12]. Evidence from mouse models suggests that RP1L1 may play a role in maintaining OS disc structure or tether the discs to the photoreceptor axoneme, as *Rp1l1* loss resulted in disorganized OSs [12]. Therefore, pathogenic variants of *RP1L1* may lead to axonemal instability, loss of OS structural integrity, and subsequent OS degeneration. *RP1L1* mutations have been reported in cone and rod disease, including OMD, *RP1L1* maculopathies, rod-cone dystrophy, and the rod degenerative disease retinitis pigmentosa (RP) [8,9,13,14].

RP is the most common inherited photoreceptor degenerative disease, characterized by peripheral vision loss and night blindness due to rod photoreceptor dysfunction and death [15]. RP is extremely genetically heterogeneous, with pathogenic mutations in many genes leading to disease. During RP progression, rod defects and degeneration lead to RPE atrophy, which causes intra-retinal pigment translocation called bone spicules. Cone degeneration occurs during late stage RP via poorly understood processes, even when the disease is caused by rod-specific genetic lesions [16].

Zebrafish are an advantageous model of photoreceptor disease, as they are diurnal vertebrates with an abundance of cone photoreceptors. Zebrafish retinas are conserved with the mammalian retina in terms of structure and function. Mice, while a commonly utilized mammalian model for human disease, are nocturnal, and due to adaptation for night activity have rod-dominant retinas with few cones and limited cone-cone interactions. Zebrafish have roughly equal proportions of cones and rods, making their retinas more similar to the human macula than other retinal regions. This allows zebrafish to be utilized for dissecting the etiology of cone and rod diseases, as well as the assessment of how rod loss impacts cone photoreceptors.

We developed a zebrafish model of *RP1L1*-associated photoreceptor disease using CRISPR/Cas9 genome editing. Mutant animals presented with progressive photoreceptor dysfunction and rod OS disorganization. During late stage disease, mutant retinas had subretinal drusenoid deposits, a feature associated with AMD.

## 2. Materials and Methods

### 2.1. Zebrafish Care

Zebrafish were maintained in the University of Alberta aquatics facility using protocols approved by the Animal Care and Use Committee: Biosciences, under guidelines from the Canadian Council of Animal Care (protocol AUP00000077). The fish were maintained at 28 °C, under a 14/10 h light/dark cycle. All control fish were of the AB line, and the mutant line was generated on the AB background. A stock solution of tricaine anesthetic was made using 400 mg of tricaine powder (Sigma Aldrich, St. Louis, MO, USA) in 97.9 mL of distilled water; 2.1 mL of 1 M Tris, pH = 9, was added, and the pH was adjusted to 7.3.

### 2.2. gDNA Extraction

The extraction of gDNA was performed as previously described [17]. NaOH at 50 mM was added to the tissue, which was boiled at 95 °C for 20 min and then cooled on ice. Tris-HCl at 1 M, pH = 8, was added to neutralize the pH, at 1/10 the volume of NaOH [17]. Samples were subsequently centrifuged, and the supernatants were isolated for DNA extraction. For larval finclips, 20 μL of NaOH was used; 50 μL was used for adult finclips, and 100 μL, for pooled embryos.

### 2.3. CRISPR/Cas9 Genome Editing

CRISPR sites were identified in the first coding exon of *rp1l1* (Gene ID: 101882236) using Geneious R9 (Biomatters Inc., San Diego, CA, USA). Previously, there were two *rp1l1* homologues annotated in zebrafish: *rp1l1a* and *rp1l1b*. However, *rp1l1b* did not resemble *rp1l1* genes in other organisms (including humans, mice, cows, pigs, dogs, chickens, and Xenopus), as it was missing defining features that identify *rp1l1* and was almost twice the size. In addition, in the current zebrafish genome (z10), *rp1l1b* is no longer annotated. Therefore, it appears that zebrafish have one *rp1l1* homolog, formerly annotated as *rp1l1a*, which we targeted for our experiments. The guide RNA (gRNA; sequence: 5′-CATCTTGACGCCTTTGAACT-3′) was synthesized as previously described [18], using the mMessage mMachine SP6 Transcription Kit (Invitrogen, Waltham, MA, USA). Embryos were injected at the single-cell stage using a glass needle mounted on a micromanipulator. The injection mix consisted of 1 μL (>1500 ng/μL) of gRNA, 2 μL of Cas9 nuclease, *S. pyogenes* (New England Biolabs, Ipswich, MA, USA), 0.5 μL of Cas9 buffer (New England Biolabs, Ipswich, MA, USA), and 1.5 μL of 1.5 M KCl.

### 2.4. Finclips and Genotyping

To select for CRISPR/Cas9-injected fish that could produce mutant offspring, mosaic P0 CRISPR-injected animals were grown to adulthood and crossed with uninjected AB fish. The 5-day post-fertilization (dpf) larval zebrafish were pooled in groups of 25 for gDNA extraction. The extracted DNA was used as a template for PCR to amplify a 479 bp fragment of *rp1l1* (forward primer: 5′-GGCTTTTTCGACGCTGATCC-3′; reverse primer: 5′-AATCCTTTTGGGGTGCCGAT-3′), which was then TOPO cloned (Invitrogen, Waltham, MA, USA) and transformed into One Shot TOP10 Chemically Competent *E. coli* cells (Invitrogen, Waltham, MA, USA). Colony PCR was performed on 10–20 colonies per larval gDNA pool, and the PCR products were purified using the QIAquick PCR Purification Kit (Qiagen, Hilden, Germany) for Sanger sequencing. Once individuals with germline mutations were identified, they were bred with AB fish, and the progeny were screened for mutations. To genotype the progeny, 3–4 dpf larvae were individually placed in a droplet of embryo medium in a plastic petri dish [19]. A sharp scalpel blade was used to remove a small piece of the tail. Single larvae were transferred to a 24-well dish with fresh embryo medium, and the tail fragment was transferred to a 1.5 mL microfuge tube using a 200 μL pipette that had PBS + 0.1% Tween aspirated into it to prevent the tissue from sticking. To isolate mutant alleles, a region of *rp1l1* was PCR amplified using the aforementioned primers, TOPO cloned (Invitrogen, Waltham, MA, USA), transformed into One Shot TOP10 Chemically Competent *E. coli* cells (Invitrogen, Waltham, MA, USA), and sequenced. Once animals with mutant alleles were isolated, they were grown and crossed with AB fish, and their progeny were in-crossed. Homozygosity for the 16-base-pair deletion allele used for this work was confirmed in adult animals through finclips, PCR amplification using the same primers, and restriction fragment length polymorphism (RFLP) using BtsIMutI (New England Biolabs, Ipswich, MA, USA).

### 2.5. qPCR

5 dpf embryos were pooled in groups of 50 and flash frozen in liquid nitrogen. RNA was extracted using the GeneJET RNA Purification Kit (Thermo Fisher Scientific, Waltham, MA, USA). Complementary DNA (cDNA) was generated using the RevertAid First Strand cDNA Synthesis Kit (Thermo Fisher Scientific, Waltham, MA, USA). The qPCR was set up with the Luminaris HiGreen High ROX qPCR kit (Thermo Fisher Scientific, Waltham, MA, USA) and performed using the 7900HT Fast Real-Time PCR System (Applied Biosystems, Foster City, CA, USA). The results were analyzed using the ΔΔCT method.

### 2.6. Electroretinography

Electroretinographic measurements were obtained as previously described [20]. Briefly, zebrafish were dark adapted for 20 min in a dim, red-illuminated room and anesthetized in a working solution of 4% stock tricaine/aquatics facility water for 2–3 min. An anesthetized fish was then transferred to a moistened polyvinyl alcohol (PVA) sponge on the testing platform and positioned on its side. The reference electrode (PN: EP08, World Precision Instruments Inc., Sarasota, FL, USA) was positioned underneath the sponge, while the Ag/AgCl recording electrode was carefully placed on the center of the cornea using a micromanipulator (Narishige International USA Inc., Amityville, NY, USA). The testing platform was then inserted into the Ganzfeld light stimulator connected to an E3 Electrophysiology System (Diagnosys LLC, Lowell, MA, USA). The testing protocol was an increasing scotopic series of 0.2, 1.0, 3.0, and 10 cd·s/m^2^ stimuli, each step consisting of 5 single flashes of white light separated by 5000 ms. A band-pass filter of 0.3–300 Hz was applied to reduce system noise. Following the testing protocol, the fish was removed from the platform and placed into a recovery tank. Zebrafish survival was maximized by gently pumping water past the gills using a 1 mL transfer pipette until normal gilling and swimming behavior was regained. Waveforms were generated by averaging 5 flashes for each stimulus intensity. The b-wave amplitude was measured as the difference in amplitude between the trough of the initial negative a-wave and the peak of the b-wave. If the a-wave was not present, the b-wave was measured from the baseline.

### 2.7. Optical Coherence Tomography

Zebrafish were anaesthetized using 4% tricaine stock solution/aquatics facility water and placed on a damp, triangular sponge in a plastic chamber, with the right eye facing upwards for imaging. The subject was secured with a strip of damp gauze with small weights on both sides and covered with tricaine/fish water solution for the procedure. The handheld Envisu R-Series OCT (Bioptigen Inc., Durham, NC, USA) was mounted perpendicularly to the zebrafish, thereby directed at the eye. The InVivoVue 2.4 OCT Management Software (Bioptigen Inc., Durham, NC, USA) was used for image acquisition and analysis. After the procedure, the animals were transferred to a recovery tank and aquatics facility water was gently passed over the gills with a 1 mL transfer pipette to resuscitate them. The fish were observed until normal swimming behavior was maintained.

### 2.8. Electron Microscopy

Zebrafish were euthanized with an 8.4% tricaine stock solution in aquatics facility water and underwent surgical cervical dislocation and enucleation. Lenses were removed with forceps, and the eyes were placed in fixative (2.5% glutaraldehyde/2% paraformaldehyde) for a minimum of 24 h before processing. Post-fixation but prior to dehydration and processing, the eyes were cut into halves to allow for the appropriate penetration of solutions. The tissue was washed with 0.1 M phosphate buffer three times for 10–15 min each, stained with 1% osmium tetroxide for 1 h, and dehydrated through an ethanol series (50%, 70%, 90%, 100%, 100%, and 100%), allowing 15–25 min for each step. The tissue was treated with a 1:1 ratio of ethanol/Spurr resin mixture for 1 h, and then, the mixture was removed and replaced with pure Spurr resin for overnight. The Spurr resin was replaced with fresh resin two more times the next day, and the tissue was subsequently embedded in resin in flat molds and cured overnight at 70 °C. Blocks were sectioned into 70–90 nm sections using a Reichert-Jung Ultracut E Ultramicrotome and placed on grids. The sections were stained with uranyl acetate for 20 min, rinsed with water, stained with lead citrate stain for 7 min, rinsed with water, dried, and imaged using a Morgagni 268 transmission electron microscope (Philips/FEI, Hillsboro, OR, USA) with a Gatan CCD camera (Gatan Inc., Pleasanton, CA, USA).

### 2.9. Paraffin Processing and Hematoxylin and Eosin Staining

Whole zebrafish heads were fixed in 4% paraformaldehyde for 48 h prior to paraffin processing. Samples were transferred into 50% ethanol for 1–3 h and then placed into a cassette and loaded into a Leica Tissue Processor 1020. The processing program steps were 1 h in 70% ethanol, 1 h in 90% ethanol, 1.5 h in 100% ethanol (twice), 1.25 h in equal parts of ethanol and toluene, 0.5 h in toluene (twice), and 2 h in wax. Tissue was embedded in paraffin blocks, sectioned with a microtome, placed on slides, and dried in a 37 °C oven.

For staining, slides were treated with toluene for 10 min to remove paraffin wax and then rehydrated using an ethanol gradient (100%, 90%, 70%, and 50% ethanol for 2 min each) and washed with distilled water. The slides were stained with Hematoxylin Gill III for 2 min, rinsed with distilled water, washed with cold tap water for 15 min, and washed with 70% ethanol for 2 min. The slides were treated with eosin for 30 s and washed twice in 100% ethanol for 2 min each, followed by two toluene washes for 2 min each. The slides were covered with Dibutyl Phthalate Xylene and a coverslip and then kept at 37 °C overnight for medium solidification. Hematoxylin and eosin (H&E)-stained sections were imaged using a ZEISS AXIO A1 Compound Light Microscope (Zeiss, Toronto, ON, Canada) with a SeBaCam 5.1MP Camera (Laxco Inc., Bothell, WA, USA).

### 2.10. Oil Red O Staining on Cryopreserved Tissue

Zebrafish eyes were fixed in 4% paraformaldehyde overnight and then dehydrated in 30% sucrose in phosphate buffer for 24 h. The sucrose was removed and replaced with Optimum Cutting Temperature compound (Fisher Scientific, Toronto, ON, Canada). Eyes were embedded, flash frozen on dry ice, and stored at −80 °C until sectioning. Tissue was then cryosectioned at 10 μm, placed on SuperFrost Plus microscope slides (Fisher Scientific, Toronto, ON, Canada), and stored at −80 °C until use.

In preparation for staining, sections were allowed to thaw at room temperature for 1 h. Tissue was treated with 4% paraformaldehyde for 20 min, washed with water, and rinsed with 60% isopropanol. A working solution of Oil Red O (0.3%) was prepared in 60% isopropanol, and the slides were stained for 15 min in a Coplin jar. The slides were rinsed with 60% isopropanol, and the nuclei were stained with hematoxylin for 1 min and rinsed with distilled water. Mount Quick aqueous mountant (Electron Microscopy Sciences, Hatfield, PA, USA) was added to the slides, coverslipped, and sealed with nail polish. Images of the sections were captured using a ZEISS AXIO A1 Compound Light Microscope (Zeiss, Toronto, ON, Canada) with a SeBaCam 5.1MP Camera (Laxco, Bothell, WA, USA).

### 2.11. Statistical Analysis

Statistical analysis was performed using Prism version 8.0 (GraphPad Software Inc., San Diego, CA, USA). For the OCT measurements, Mann–Whitney U tests were performed; for the electroretinography (ERG) results, unpaired T-tests were used to compare the genotypes and repeated-measures ANOVAs with Tukey post hoc comparison to investigate differences within the groups. The qPCR data were analyzed using the ΔΔCT method, and statistical analyses were performed on the ΔΔCT values using Mann–Whitney U tests. The propagation of error was calculated to determine the upper and lower limits of error.

## 3. Results

### 3.1. Generation of *rp1l1* Mutant Zebrafish

**Figure 1:**
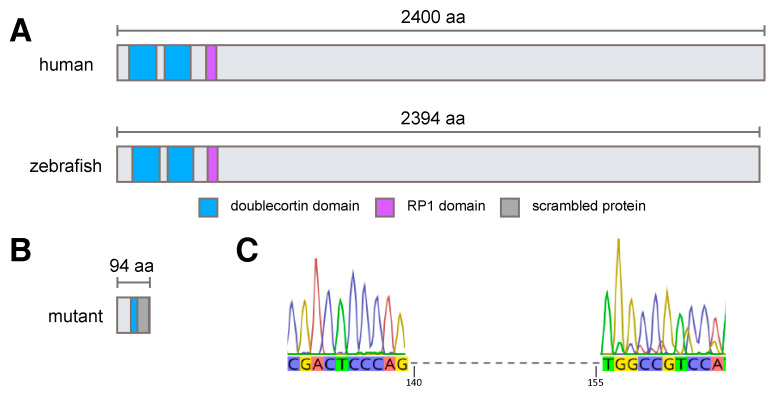
Human and zebrafish RP1L1 proteins have conserved domains, which are predicted to be eliminated in *rp1l1* mutant zebrafish. (**A**) Cartoon of human and wild-type zebrafish proteins. Human and zebrafish RP1L1 have conserved domains and are similar sizes, with human RP1L1 protein being 2400 amino acids (aa) and zebrafish Rp1l1 protein being 2394 amino acids in length. (**B**) The mutation in zebrafish *rp1l1* is predicted to result in a severely truncated, nonfunctional Rp1l1 protein. The mutation occurs in the first doublecortin domain of Rp1l1 after amino acid 52, resulting in significant loss of more than half of the doublecortin domain, which is followed by an abnormal protein extension and termination at 94 amino acids in length. (**C**) The CRISPR/Cas9-generated 16 bp deletion in zebrafish *rp1l1* occurs after nucleotide 139.

Human RP1L1 is a 2400 amino acid protein with two doublecortin (microtubule-binding) domains and an RP1 domain towards the N-terminus. Zebrafish Rp1l1 is similar in size, at 2394 amino acids in length (Figure 1), with the most well conserved sequences in the doublecortin and RP1 domains (Figure S1). As the most frequent OMD-causing mutation occurs in the first doublecortin domain of human RP1L1 and this region is well conserved between the species, we targeted the homologous region in zebrafish *rp1l1* using CRISPR/Cas9 genome editing. We generated a 16 base pair (bp) deletion in the first doublecortin domain of *rp1l1* (Figure 1C). This deletion is predicted to severely truncate the Rp1l1 protein and abolish all functional domains (Figure 1B, Figure S1). Homozygous mutant zebrafish were viable, fertile, and had no overt ocular defects at any developmental stage.

To assess whether the mutant *rp1l1* transcript underwent nonsense-mediated decay, we utilized qPCR. The relative abundance of the *rp1l1* transcript was not decreased in mutant zebrafish (average of 2.36-fold increase, lower limit = 1.6, upper limit = 3.4; n = 4). The mutant transcript was not obviously lost via nonsense-mediated decay, but the frameshift mutation in the transcript is predicted to encode a nonfunctional Rp1l1 protein lacking all recognizable domains (Figure 1).

### 3.2. *rp1l1* Mutants Have Progressive Photoreceptor Dysfunction

Functional assessment of the photoreceptors was performed with electroretinography (ERG) under scotopic (dark-adapted) conditions. An amplitude reduction in the ERG waveforms suggests an inability of the photoreceptor to appropriately respond to the light stimulus and transmit information to downstream neurons. The light stimulus intensities used for the ERG testing were 0.2 cd·s/m^2^, which elicits a rod-dominated response, and 1, 3, and 10 cd·s/m^2^, which elicit a mixed rod–cone response. Figure S2 shows repeated-measures analysis for WT and *rp1l1* mutants for the ERG intensities.

**Figure 2:**
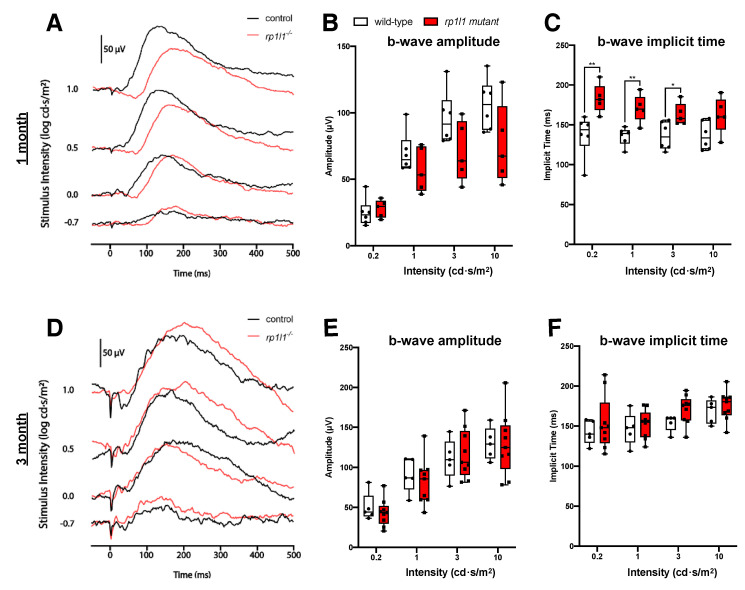
*rp1l1* mutants have photoreceptor responses comparable to wild-type zebrafish at 1 and 3 months. (**A**) Sample traces from one mutant and one wild-type animal at 1 month of age. (**B**) b-wave amplitude is not different between wild-type and mutant fish. (**C**) 1-month-old mutant zebrafish have increased b-wave implicit time under 0.2, 1, and 3 intensities, indicating a delay in response. n = 6 for wild-type; n = 5 for mutant. (**D**) Sample traces from a 3-month-old wild-type and mutant animal. (**E**) b-wave amplitude and (**F**) implicit time are not significantly different between the groups. The box and whisker plots depict medians (center lines), upper and lower quartiles (boxes), and distributions (bars). Each dot is an individual animal. n = 5 for wild-type; n = 9 for mutants. * *p* < 0.05; ** *p* < 0.01.

1 month (juvenile) and 3 months old (young adult) mutant animals had scotopic b-wave amplitudes comparable to wild-type (WT) under all intensities tested (Figure 2), suggesting photoreceptor function comparable to WT. However, the b-wave onset was significantly delayed in the 1-month-old mutants for the 0.2, 1, and 3 cd·s/m^2^ intensities (Figure 2C); this recovered by 3 months of age (Figure 2F). Taken together, the ERG assessment suggests that young mutant animals have functional photoreceptors.

**Figure 3:**
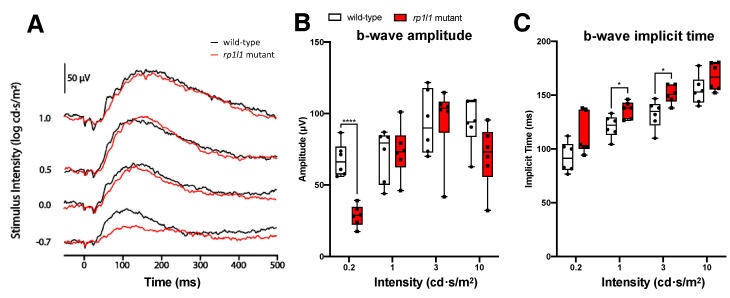
*rp1l1* mutants have reduced dim light responses at 6 months. (**A**) Sample traces from a wild-type and an *rp1l1* mutant zebrafish at 6 months old. (**B**) b-wave amplitudes for wild-type and mutant groups. Mutant animals have a reduced b-wave amplitude for the dim light stimulus (0.2 cd·s/m^2^ intensity) but normal amplitudes under brighter intensities. (**C**) Implicit time for the b-waves at the tested intensities. The mutants had delayed b-waves for the 1 and 3 cd·s/m^2^ intensities. The box and whisker plots depict medians and distributions, with all animals plotted. n = 6 for both groups. * *p* < 0.05; **** *p* < 0.0001.

At 6 months of age, mutant animals had significantly decreased b-wave amplitudes when exposed to the 0.2 cd·s/m^2^ intensity stimulus; the average b-wave amplitude was decreased by 57.9%. Responses were comparable to WT under all other intensities (Figure 3). The reduced dim stimulus amplitude indicates that 6-month-old *rp1l1* mutants had defects in rod photoreceptors. Intriguingly, the b-wave implicit time was increased for the 1 and 3 cd·s/m^2^ intensities in the mutants, suggesting that the photoreceptors were not able to transmit information at a typical rate for those intensities (Figure 3C).

**Figure 4:**
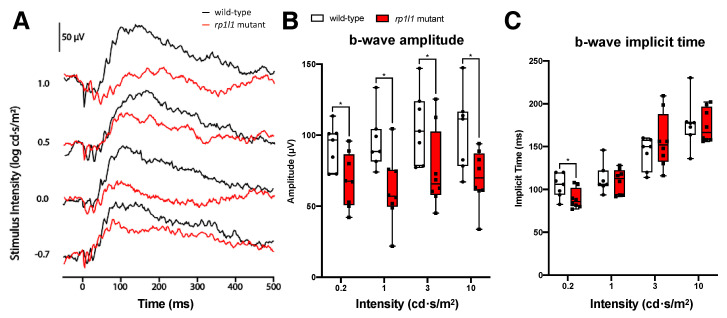
*rp1l1* is required for the normal photoreceptor physiological response in aged animals. (**A**) Sample traces from a 12-month-old wild-type and *rp1l1* mutant fish. (**B**) b-wave amplitude for wild-type and *rp1l1* mutants. The *rp1l1* mutant zebrafish have a significantly decreased b-wave amplitude under all light intensities. (**C**) b-wave implicit time for the wild-type and mutant zebrafish. The mutants have a significantly faster b-wave under the 0.2 cd·s/m^2^ intensity. The box and whisker plots depict medians and distributions, with all animals plotted. n = 7 for wild-type; n = 8 for mutants. * *p* < 0.05.

12-month-old mutant zebrafish had significantly decreased responses under all light intensities tested (Figure 4). The average b-wave amplitudes for the mutants were decreased by 25.8% for the 0.2 cd·s/m^2^ intensity stimulus, 35.7% for the 1 cd·s/m^2^ intensity, 28.7% for the 3 cd·s/m^2^ intensity, and 31.5% for the 10 cd·s/m^2^ intensity. The decrease in b-wave amplitude at higher intensities was not observed at 6 months, and suggests progressive rod deficits in the *rp1l1* mutants. Mutants had significantly faster b-wave onset than WT for the 0.2 cd·s/m^2^ intensity stimulus but not the other intensities (Figure 4C).

Taken together, these functional assessments suggest that *rp1l1* mutant zebrafish have progressive photoreceptor dysfunction. In light of this, we focused further investigations on aged *rp1l1* mutant zebrafish.

### 3.3. Live Imaging Reveals Abnormalities in Outer Retinas of *rp1l1* Mutant Zebrafish

**Figure 5:**
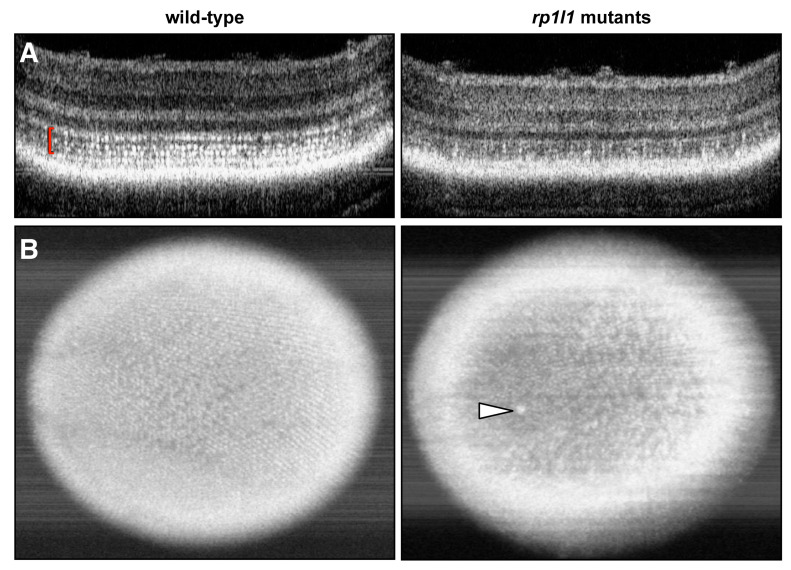
*rp1l1* mutant zebrafish have fewer hyper-reflective inner segment punctae and gaps in the photoreceptor mosaic. Optical coherence tomography (OCT) of wild-type and *rp1l1* mutant zebrafish retinas at 12 months. (**A**) OCT B-scans through zebrafish retinas. In the wild-type animals, the photoreceptor inner segments (seen as hyper-reflective dots, red brackets) are abundant and uniformly distributed. In mutant animals, this part of the retina is very patchy and appears to have fewer punctae. (**B**) En face projections through the retina. In the mutant animals, the mosaic appears patchy and disrupted. There is also a large hyper-reflective speck in the retina of the mutant animal (arrowhead), which typically indicates an immune cell response.

We assessed retinal structure in live animals to investigate how the functional deficits related to retinal architecture in aged zebrafish using OCT. Photoreceptor inner segments (ISs) are highly reflective due to the abundance of densely packed mitochondria [21] and appear as bright punctae on zebrafish OCT. The hyper-reflective punctae form banding patterns due to the different heights of the photoreceptor subtypes, and we refer to this area as the IS/OS region, as it contains both ISs and OSs. In WT animals, the hyper-reflective punctae in the IS/OS region are numerous and form the expected banding pattern (Figure 5A). However, 12-month-old mutant zebrafish had visibly fewer punctae on sectional OCT scans (Figure 5A). This was similarly observed in en face projections, which provide a view of the photoreceptor mosaic. The WT en face projections show a full mosaic with clearly distinguishable rows of photoreceptors (Figure 5B). Mutant en face images have fewer punctae and the rows are difficult to distinguish (Figure 5B). Of note, some of the mutants also had large hyper-reflective blebs in their OCT images, characteristic of activated immune cells that have translocated to the outer retina [22,23,24,25]. Immune cell translocation is commonly seen in response to photoreceptor disease or outer retinal injury [22,25,26,27].

**Figure 6:**
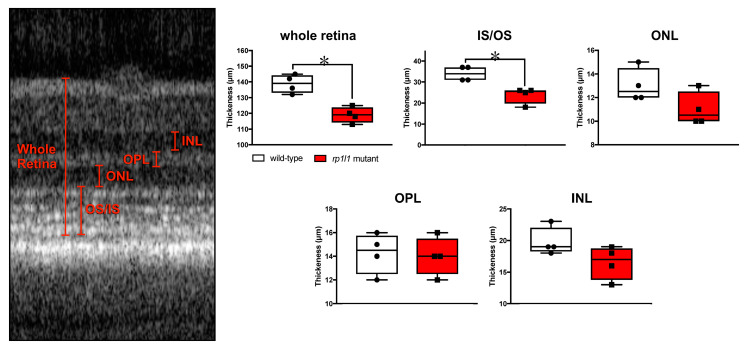
*rp1l1* mutant zebrafish have thin retinas. Measurements of retinal thickness from OCT scans of 12-month-old wild-type and *rp1l1* mutant zebrafish. Measured regions are shown on the OCT scan (left). Mutants have statistically significantly thinner retinas and IS/OS areas. Other regions were not statistically different. The box and whisker plots depict medians and distributions, with all animals plotted. n = 4/group; * *p* < 0.05; IS = inner segment; OS = outer segment; ONL = outer nuclear layer; OPL = outer plexiform layer; INL = inner nuclear layer.

Next, we measured the retinal layer thickness in the OCT images (Figure 6). The mutant animals had significantly thinner IS/OS regions, which contributed to thinner retinas overall (Figure 6). Measurements were also taken for the outer nuclear layer (ONL), where the photoreceptor cell bodies are found; the outer plexiform layer (IPL), where the photoreceptors synapse with downstream interneurons; and the inner nuclear layer (INL), where the interneuron cell bodies reside. These retinal regions were not statistically different in measurement between the WT and mutant animals, although the ONL and INL trended towards a decrease in the mutants. The lack of statistically significant differences between the genotypes for ONL and INL thicknesses may be attributable to modest sample size. These findings suggest that the photoreceptor IS/OS region is disrupted in the mutant animals, but that the cell bodies may remain intact. Taken together, the OCT results show that the mutant animals had abnormal photoreceptors at 12 months of age.

### 3.4. *rp1l1* is Required for Normal Organization of Photoreceptor Outer Segments

**Figure 7:**
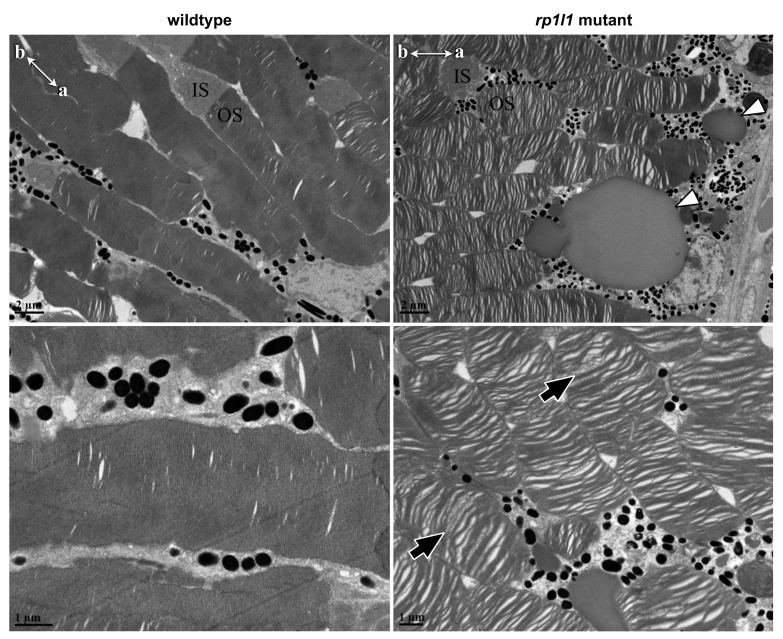
*rp1l1* mutant zebrafish have disorganized photoreceptor outer segments and deposits between the photoreceptors and retinal pigment epithelium. Electron microscopy of 11-month-old mutant and wild-type photoreceptors. Mutant photoreceptors appear disorganized, with gaps between the discs, and discs wave or swirl in some outer segments (black arrows). Additionally, there appear to be deposits between the photoreceptor outer segments and retinal pigment epithelium (white arrowheads). IS = inner segment; OS = outer segment. Compasses in top-left corners show apical (a) and basal (b) photoreceptor orientations for the images.

As Rp1l1 is a structural component of the photoreceptor OS, we assessed how OS morphology was impacted in our *rp1l1* mutant zebrafish. WT OS discs observed by electron microscopy (EM) were densely packed, with few visible spaces between them, and the discs uniformly spanned the OS width (Figure 7). EM of 11-month-old zebrafish retinas showed disorganized photoreceptor OSs, with many gaps and uneven, swirling discs (Figure 7). The vast majority of the photoreceptor OSs were disorganized in mutant retinas, and few OSs had uniformly organized discs throughout the whole OS. In some OSs, we observed regions of normal appearing OS disc organization next to disorganized areas. The regions of normally organized OS discs were often at the most apical tip of the OS in distally positioned photoreceptors, which are most likely rods.

### 3.5. Diseased Zebrafish Retinas Have Lipid-Rich Subretinal Drusenoid Deposits

**Figure 8:**
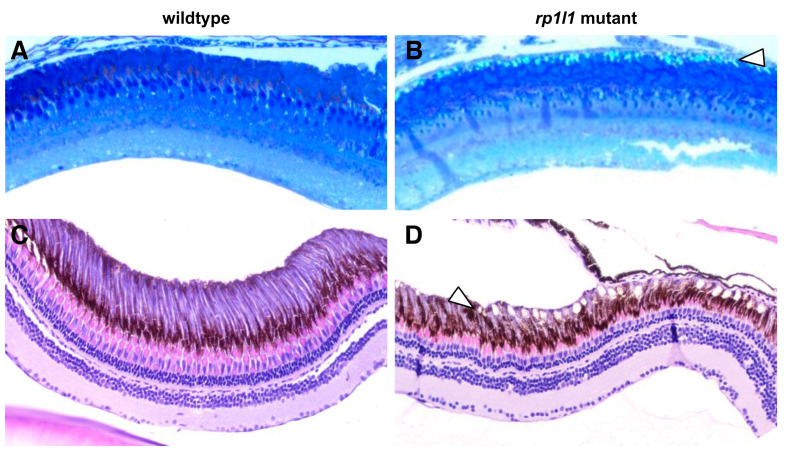
Mutant zebrafish retinas have subretinal deposits and short outer segments. Wild-type and mutant retina sections, stained with azure blue (**A**,**B**) or hematoxylin and eosin (H&E) (**C**,**D**). Deposits can be observed in the 11-month-old mutant azure blue-stained (**B**) sections between the photoreceptor outer segments and retinal pigment epithelium (arrowheads). H&E-stained sections of 12-month-old mutant retinas (**D**) show similar accumulations (arrowhead); however, the material that had filled the deposits was stripped from the tissue during processing. *rp1l1* mutant retinas have shorter outer segments and thinner outer nuclear layers (**D**). No deposits were observed in the wild-type retinas (**A**,**C**).

**Figure 9:**
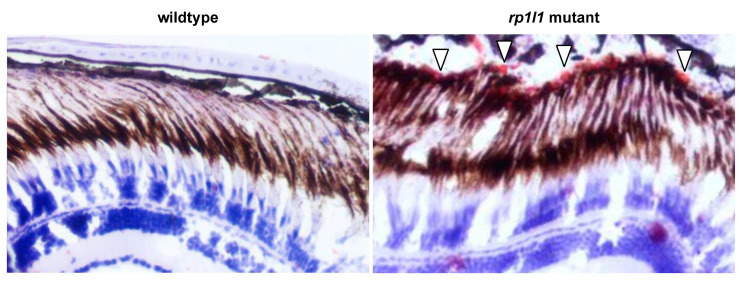
*rp1l1* mutants have lipid-rich subretinal drusenoid deposits. Cryosections of 14-month-old wild-type and mutant zebrafish retinas, treated with Oil Red O to stain lipids and hematoxylin to stain nuclei (blue). The deposits in the mutant retina stained red (arrowheads), demonstrating that they are lipid-rich.

We observed deposits between the photoreceptor OS and RPE in the EM images of mutant retina (Figure 7). The deposits were globular and relatively small—typically 3–10 μm in diameter. EM thick sections stained with azure blue also showed deposits under light microscopy (Figure 8A,B). H&E staining similarly showed accumulations between the OSs and RPE, as well as short OSs and visibly thinner outer nuclear layers, in 12-month-old animals (Figure 8C,D). On H&E stained sections, but not the EM thick or ultrathin sections, the deposits appeared to be empty holes, suggesting that paraffin processing stripped the contents of the accumulations out of the tissue. Paraffin processing is known to have such an effect on lipid-rich materials. To assess whether these subretinal deposits in the mutant animal retinas were indeed lipid-rich, we next used Oil Red O staining. We found that in 14-month-old animals, the retinal deposits became stained with Oil Red O, indicating that they were lipid-rich (Figure 9). The location and positive staining for lipids suggests that these deposits are comparable to subretinal drusenoid deposits observed in human patients. No such deposits were observed in WT zebrafish retinas using any of the described methods.

## 4. Discussion

Animal models that can provide insight into photoreceptor disease and drusen formation are urgently needed to allow for the development and characterization of therapeutic strategies to prevent vision loss. We report a novel model of *RP1L1*-associated photoreceptor disease that has progressive photoreceptor dysfunction, seemingly caused by structural abnormalities at the level of the photoreceptor OS. In a previous study, the knockdown of *rp1l1* was performed in larval zebrafish, but the authors did not characterize photoreceptor function or retinal features [28]. Our study provides novel insight into the function of *rp1l1* in the aging zebrafish retina. To our knowledge, this is the first report of a zebrafish model of photoreceptor disease exhibiting subretinal drusenoid deposits. Photoreceptor defects similar to what is observed in RP and AMD-like lipid deposits in this model provide the opportunity to investigate the means by which photoreceptors can be preserved and how lipid accumulations develop and impact the diseased retina. Additionally, the cone-rich zebrafish retina will allow for the assessment of how drusenoid deposits influence cones and how cones respond to rod dysfunction.

Electrophysiological assessment revealed progressive photoreceptor dysfunction in the *rp1l1* mutant zebrafish. The b-wave amplitude was comparable to WT in 1- and 3-month-old animals, abnormal under the dimmest intensity at 6 months, and abnormal under all light intensities at 12 months of age. However, we observed a delay in the b-wave implicit time at 1 month that recovered by 3 months. The cause and functional impact of the delayed b-wave in 1-month-old *rp1l1* mutants is difficult to interpret. The recovery of the b-wave implicit time by 3 months may be due to changes in photoreceptor proportions, organization, and retinal circuitry. At 1 month, zebrafish photoreceptors are primarily organized in a larval mosaic, which has many more cones than rods at a ratio of ~4:1 [29,30,31,32]. At 3 months of age, the photoreceptors are organized in a row mosaic, with roughly equal proportions of cones to rods [29]. These differences between the ages may result in the b-wave implicit time changes observed in the mutants. Interestingly, at 12 months, after photoreceptor dysfunction progressed to impact photoreceptors under all tested light intensities, b-wave onset was increased in speed for the dimmest intensity. Why this was observed and what impact a shorter implicit time would have on the photoreceptors is unknown and requires further study.

The function of RP1L1 protein is unknown. However, evidence from mutant mice suggests that it is involved, either directly or indirectly, in maintaining OS morphology; *Rp1l1* mutant mice had progressive photoreceptor degeneration, thinner outer retinas, and subsets of disorganized OSs with wavy discs [12]. As RP1L1 is a microtubule-associated protein that localizes to the photoreceptor axoneme, it may be tethering the outer segment discs to the cilium core through its interaction with protein binding partners [8,10,12]. We observed disorganized photoreceptor OSs in our *rp1l1* mutant zebrafish. This observation coincides with data from *Rp1l1*-deficient mice [12]; however, only a subset of photoreceptor OSs in *Rp1l1* mutant mice were disorganized [12], whereas the majority of OSs in our mutant zebrafish were affected. Interestingly, some photoreceptors in our mutant zebrafish had disorganized discs through the length of the OS, except for a small stretch of discs at the apical tip that appeared normally organized. The axoneme does not extend through the entire rod photoreceptor OS, and the discs in the most apical OS region may be organized by RP1L1-independent means.

RP1L1 has a synergistic role with RP1, another photoreceptor-specific component of the axoneme that shares many features with RP1L1 [12,33,34]. RP1L1 and RP1 proteins are comparable in terms of size, domains, and localization, and both result in OS disorganization when mutated in animal models [12,34,35]. *RP1L1* mutations have been reported to cause a diversity of human photoreceptor diseases that can affect cones and rods [8,9,13], while *RP1* mutations have primarily been reported in retinitis pigmentosa cases, although there have been some cone diseases associated with *RP1* [36,37,38]. RP1L1 likely has unique roles or binding partners that it does not share with RP1 and may have functions specific to cones or rods. Specialized subfunctions for RP1L1 in different photoreceptor types could explain why *RP1L1* mutations can cause both cone and rod disease, in a mutation-specific manner.

We report the accumulation of subretinal drusenoid deposits between the photoreceptor OS and RPE in the mutant animals. Subretinal drusenoid deposits and drusen are common in aging humans and are defining features of AMD, but how they impact retinal disease is not known [39]. Subretinal drusenoid deposits (also called reticular drusen, reticular pseudo-drusen, or reticular lesions) are a newly characterized feature of aging and AMD and are therefore not as well investigated as drusen [39,40,41]; however, the presence of subretinal drusenoid deposits has been reported to more than double an individual’s likelihood of developing AMD [39]. AMD patients can have both subretinal drusenoid deposits and drusen, but may alternatively have one and not the other. Drusen most frequently develop in the cone-rich central macula in humans, while subretinal drusenoid deposits typically occur in the comparatively rod-rich outer macula, though this is not always the case. These trends suggest that the photoreceptor types influence the development of drusen and subretinal drusenoid deposits. Thus, drusen and subretinal drusenoid deposits may be infrequently reported in small animal models due to the photoreceptor types present and their organization.

Small animal models of drusen and drusen-like deposits have been sought after for decades. There are some mouse models that develop drusen similar to what is observed in humans in terms of composition and location [42,43,44,45]. Recently, a mouse model with photoreceptor dysfunction and subretinal drusenoid deposits due to impaired cholesterol metabolism was reported [46]. However, some presumed models of drusen unfortunately had deposits that differed greatly from drusen observed in the human retina. In some cases, murine drusen-like accumulations were expanded macrophages rather than lipids [47]. Additionally, white lesions observed on funduscopy appeared drusen-like but were actually indicators of retinal atrophy or intra-retinal lesions, not lipid accumulation [48,49,50]. Of note, mouse models that develop drusen or subretinal drusenoid deposits are often complex double mutants or have mutations in genes not associated with AMD [42,43,44,45,46]. Our zebrafish model of photoreceptor disease in a cone-rich retina has mutations in a gene known to cause photoreceptor degeneration in humans. Prior to our study, whether diseased zebrafish retinas could develop subretinal drusenoid deposits was unknown.

The origin of drusen and drusenoid deposits is not well understood. Drusen and drusenoid deposits may both result from RPE cell metabolic dysfunction in a diseased retina. As drusen develop between the choroidal vasculature and the RPE, it has been postulated that drusen may be derived from choroid-deposited materials. Indeed, the protein components of drusen are typically found in the blood or RPE [51]. However, the blood–retina barrier protects the retina from leakage of substances from the blood into the eye, and this hypothesis alone does not explain the components of drusen that seem RPE-derived. It is possible that in the healthy retina drusen-forming materials are normally deposited by the choroid and then taken up and broken down by the RPE, but that with age and disease, the RPE loses cells or becomes dysfunctional, resulting in ineffective RPE phagocytosis and accumulation of deposited materials. In contrast, drusenoid deposits are found between the OS and RPE, making a vascular origin much less likely. There is evidence that regression of subretinal drusenoid deposits can coincide with severe outer retinal thinning and loss of photoreceptor OSs in AMD patients [52]; this could suggest that photoreceptor OSs, or OS interaction with the RPE, is key for the development of subretinal drusenoid deposits. Drusenoid deposits contain some of the same components as drusen, including complement factor H, vitronectin, and ApoE [40]. The drusenoid deposits observed in our model provide the opportunity to investigate the cellular origins and protein composition of drusenoid deposits. This is an important finding, as animal models rarely develop drusen or drusen-like deposits. Our model may be relevant to AMD by providing the unique opportunity to study subretinal drusenoid deposits in photoreceptor disease progression, and how this impacts photoreceptor and RPE health and function over time.

Photoreceptor degeneration is an irreversible cause of vision loss and blindness. Investigating the disease mechanisms and genetic factors that impact disease onset and progression is essential for combating vision loss and generating effective therapies for patients. *RP1L1* mutations underlie a spectrum of photoreceptor disease, and treatments for *RP1L1*-associated conditions cannot be developed without a keen understanding of the function of RP1L1 in photoreceptors. Our model of *RP1L1* photoreceptor disease provides the opportunity to investigate the role of RP1L1 in photoreceptor degeneration as well as the formation and impact of subretinal drusenoid deposits in the retina.

## Supplementary Materials

The following are available online at https://www.mdpi.com/2073-4409/9/10/2214/s1. Figure S1: CRISPR/Cas9-induced mutation in zebrafish *rp1l1*. (**A**) Alignment of human RP1L1 (top) and zebrafish Rp1l1 (bottom) proteins, showing sequence conservation in the doublecortin and RP1 domains. (**B**) The CRISPR/Cas9-induced 16 bp deletion in the first coding exon of rp1l1 in our mutant zebrafish. (**C**) Location of the deletion in the Rp1l1 protein and its predicted consequence. The mutation results in a scrambled protein sequence after amino acid 52 and a premature stop codon after amino acid 94. Figure S2: Differences between stimulus responses for b-wave amplitude and implicit time for each group, analyzed using repeated-measures ANOVA. Wild-type in white; mutants in red. * *p* < 0.05; ** *p* < 0.01; *** *p* < 0.001; **** *p* < 0.0001.
