# Supplementary material for: Progressive Photoreceptor Dysfunction and Age-Related Macular Degeneration-Like Features in rp1l1 Mutant Zebrafish
Source: Cells. 2020 Sep 30;9(10):2214. doi: 10.3390/cells9102214 (PMC7600334; doi:10.3390/cells9102214)
Supplement: Supplementary file 1 [file cells-09-02214-s001.pdf]

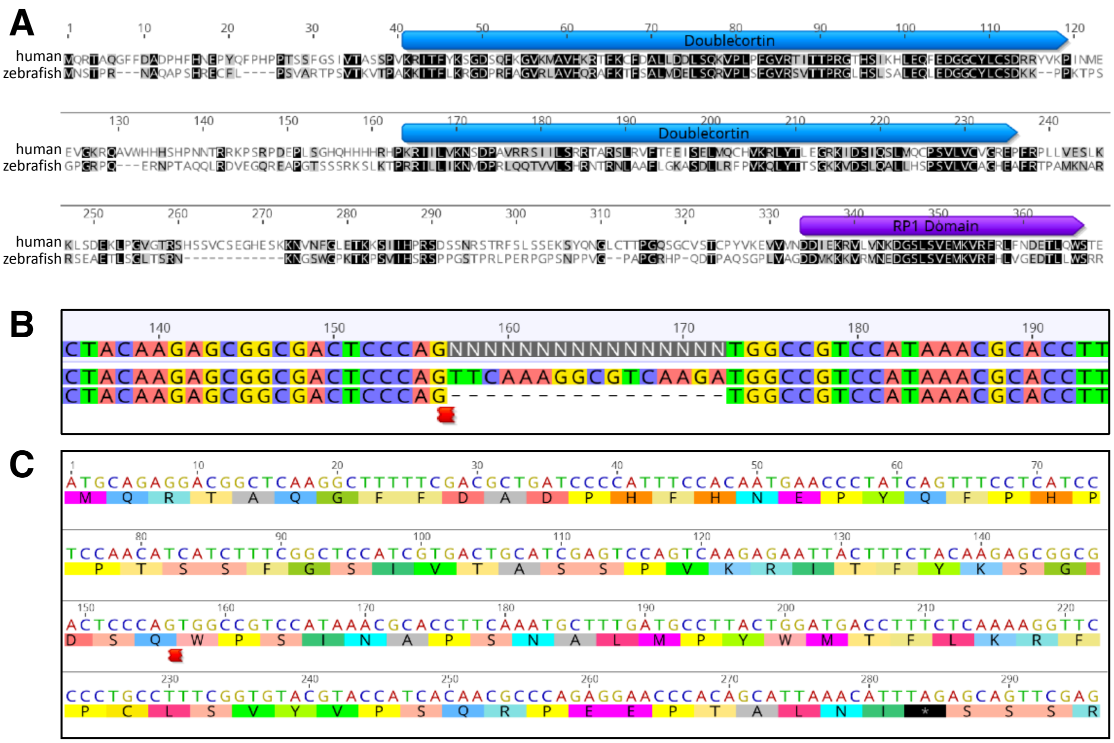

Figure S1: CRISPR/Cas9-induced mutation in zebrafish *rp1l1*. (**A**) Alignment of human RP1L1 (top) and zebrafish Rp1l1 (bottom) proteins, showing sequence conservation in the doublecortin and RP1 domains. (**B**) The CRISPR/Cas9-induced 16 bp deletion in the first coding exon of rp1l1 in our mutant zebrafish. (**C**) Location of the deletion in the Rp1l1 protein and its predicted consequence. The mutation results in a scrambled protein sequence after amino acid 52 and a premature stop codon after amino acid 94.

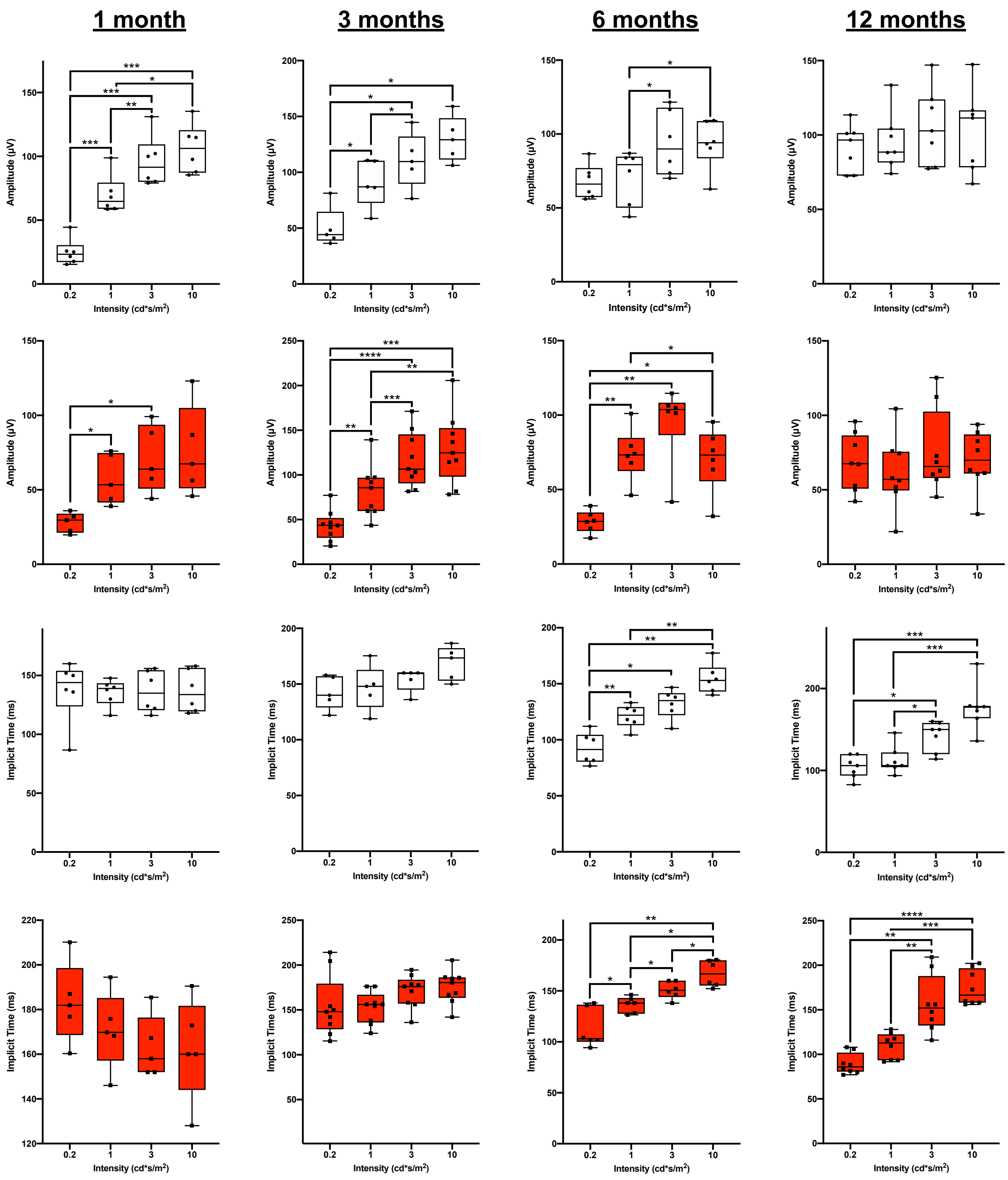

Figure S2: Differences between stimulus responses for b-wave amplitude and implicit time for each group, analyzed using repeated-measures ANOVA. Wild-type in white; mutants in red. *p < 0.05; **p < 0.01; ***p < 0.001; ****p < 0.0001.
